# With Great Power Comes Great Responsibility—A Personal Philosophy for Communicating Science in Society

**DOI:** 10.1523/ENEURO.0200-16.2016

**Published:** 2016-09-08

**Authors:** E. Paul Zehr

**Affiliations:** 1 Rehabilitation Neuroscience Laboratory, University of Victoria, Victoria, British Columbia V8P SC2, Canada; 2 Human Discovery Science, International Collaboration on Repair Discoveries (ICORD), Vancouver, British Columbia V5Z 1M9, Canada; 3 Centre for Biomedical Research, University of Victoria, Victoria, British Columbia V8P 5C2, Canada; 4 Division of Medical Sciences, University of Victoria, British Columbia V8P 5C2, Canada; 5 School of Exercise Science, Physical, & Health Education, University of Victoria, British Columbia V8P 5C2, Canada; 6 Zanshin Consulting Inc., Victoria, British Columbia V8P 5C2, Canada

## Abstract

Many think that communicating science is a necessary and rewarding activity. Yet finding compelling, relevant, and timely points of linkage between challenging scientific concepts and the experiences and interests of the general public can be difficult. Since science continues to influence more and more aspects of daily life and knowledge, there is a parallel need for communication about science in our society. Here I discuss the **“**middle-ground hypothesis**”** using popular culture for science communication and applying the **“**FUNnel model,**”** where popular culture is used as a lead**-**in and wrap**-**up when discussing science. The scientific knowledge we find in our hands does not belong to us—we just had it first. We can honor that knowledge best by sharing it as widely as possible using the most creative means at our disposal.

## Significance Statement

Using popular culture in science communication can allow the sharing of knowledge to the largest audience.

“Most of the fundamental ideas of science are essentially simple, and may, as a rule, be expressed in a language comprehensible to everyone.”

-Albert Einstein

“…With great power there must also come—great responsibility!”

-Stan Lee

## There and back again: a neuroscientist’s epiphany

Back in 2005 I began to question the broader societal impact of my work as a neuroscientist. At that time, my most heavily cited paper had ∼150 citations, and, although I realized this was a harsh interpretation, I asked myself, what if that number meant that only 150 people read my paper? Was 150 readers an acceptable impact for me in the “traditional” academic sense? My answer then and now—that same paper has over 400 citations and my body of work ∼4000 total citations—was “no.” I decided to make conversations with the general public, in addition to the community already engaged in academic literature, a stronger emphasis in my activities.

Since that time, I have been involved in many “outreach” activities focused on a general public audience—writing books, blogs, media, and talks for science promotion. I have also worked to encourage my students and colleagues to get more involved in science communication. When I compare other attempts to quantify impact in my communication activities, they contrast sharply in numbers. For example, my blog at *Psychology Today* magazine has over 250,000 page views.

Many think that communicating science to the public is a necessary and rewarding activity; however, finding compelling, relevant, and timely points of linkage between challenging scientific concepts and the experiences and interests of the general public can be difficult. Despite these challenges, science continues to influence more and more aspects of daily life as knowledge and communication about science continue to increase in necessity and importance in our society. In his amazing book “The Demon Haunted World—Science as a Candle in the Dark,” the late Carl Sagan wrote about the lack of understanding of science and described it as “…a prescription for disaster…sooner or later this combustible mixture of ignorance and power is going to blow up in our faces” (Sagan, 1995).

There are many problematic consequences of a society at large that remains without scientific knowledge, has little understanding of the scientific process, or feels segregated from the concepts. For example, elected officials may not fight for and lobby for providing funding for research and may be elected on platforms based on gross inaccuracies and flawed logic. Others include the antivaccine movement, and concussion in children’s activities and their long-term impact.

This commentary is based mostly on my own experiences using icons in popular culture to serve as vehicles for communicating science. For example, I used the Walking Dead to illustrate human motor control in a zombie context (Zehr and Norman, 2015), and Darth Vader to consider phantom limbs, embodiment, and neural prosthetics (Zehr, 2015a). The bulk of my work in this area, though, has been to use superheroes. These efforts have also led to advances in my own approach to undergraduate education. At the University of Victoria, I now teach a 100 level course **“**The Science of Batman**”** that is open to students from all faculties and departments with an interest in science and superheroes.

I explored themes of plasticity in biological systems in **“**Becoming Batman: The Possibility of a Superhero**”** (Zehr, 2008), and **the** enhancement of biological function with technology in **“**Inventing Iron Man: The Possibility of a Human Machine**”** (Zehr, 2011b). Here, I will not talk much about the need for Science Committee—please see David Eagleman’s essay on its importance (Eagleman, 2013). Instead, the focus is largely on examples of science communication using pre-existing elements in society and popular culture.

Previously, I outlined some of my early efforts (Zehr, 2011a) and advanced the “middle-ground hypothesis” using popular culture for science communication (Zehr, 2014a). Here, I extend that concept and describe some strategies that form the central core of my philosophy of science communication. To resonate with this colloquial approach, the structure of this commentary is deliberately written in a journalistic style using the first person voice.

## Understanding the needs of your audience is the key to effective science communication

Science often makes audiences uncomfortable because it forces them outside their base of knowledge. So, I try to make things as pleasant—and as fun—as I can. This maximizes the likelihood of my audience engaging with the science concepts I have chosen because they want to do so. To facilitate this engagement, I use popular culture as the link between science and the general public. Taking something the audience is familiar with (e.g., superheroes) and linking it with something they are not (e.g., neuroplasticity) allows them to enter a conversation without putting up barriers.

Although there can be many other approaches, I strongly encourage using popular culture because it is, as the name says, already popular. Superhero movies and television shows continue to have extreme popularity and represent excellent opportunities for exploring scientific concepts in a middle-ground mental “landscape” that is comfortable and familiar.

Communications guru Marshall Mcluhan said that “the medium is the message” (McLuhan, 1964) to highlight the importance of both knowledge and the manner in which it is presented. We must combine the medium and our message to truly communicate with our audiences. I strongly urge the use of ready-made vehicles, such as popular culture icons, because they represent the most seamless access to the interests of the general public. For me, superheroes are perfect for this since they afford well known examples of exploring the truth and fiction of science that underlie their fictional powers.

**Figure 1.**
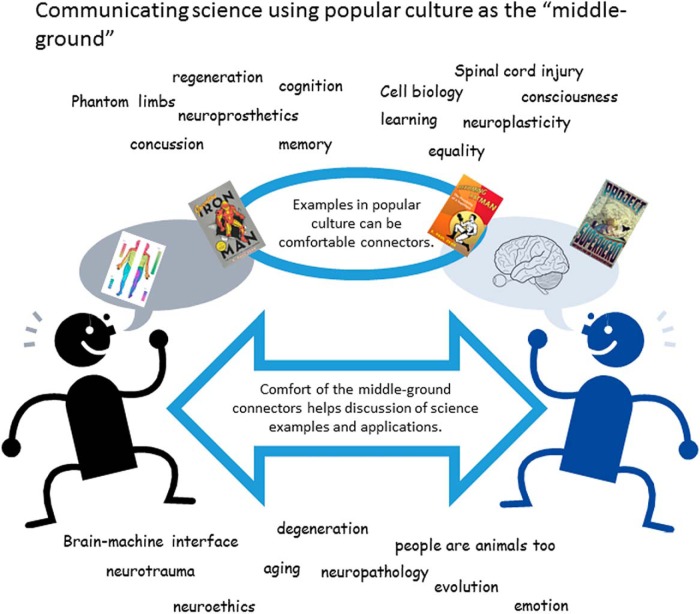
Communicating science using popular culture as the “middle ground.” This basic illustration shows the idea of a conversation between scientists and the general public. Information is exchanged using the bridging afforded by popular culture icons in the “middle ground.” The words and phrases found around the figures represent science concepts used in this way in the books “Becoming Batman,” “Inventing Iron Man,” and “Project Superhero.” In the process of writing these books and selecting concepts for discussion, I consulted “Neuroscience Core Concepts: The Essential Principles of Neuroscience” (Society for Neuroscience, 2016) and core principles in physiology (Michael et al., 2009).

In the approach I favor, the medium becomes the middle ground for the message. Popular culture as both medium and content becomes the connecting point between the science and the audience. This middle-ground hypothesis is shown in Figure 1 and is meant to apply widely to communication among all groups and ages. This illustration shows how a common middle ground can facilitate the movement of ideas. Around the folks shown talking together are science concepts that I have addressed in my books and blogs using superhero popular culture icons.

## Some examples of using popular culture as the middle ground

 In “Becoming Batman,” I used the well known superhero Batman to represent the ultimate human produced by physical and mental training (Zehr, 2008). The key point was for readers to better understand their own bodily function while thinking about that of Bruce Wayne. To evaluate what parts of Batman’s mythology might or might not be grounded in science, I surveyed neuroscience, genetics, biomechanics, psychology, physiology, and pathological outcomes. This involved comparing the representation of Batman’s skills and abilities shown in comics, graphic novels, TV, and movies to what might be found in real occupations that contain those same elements.

The result suggests that DC Comics’ Dark Knight is a mix of a NASCAR driver, NFL running back, mixed martial artist, Parkour expert, and Cirque du Soleil gymnastic acrobat. Importantly, acquiring the skills and abilities necessary for all these activities means Batman is also subjected to all the associated physical stresses and strains that produce negative outcomes in the form of injury and illness.

Using this popular culture middle ground scaffolding I addressed the many scientific components and concepts underlying the adaptations that would be needed to actually produce the Caped Crusader. The overarching concept was to view all of Batman’s training and actions as challenges to homeostasis and balance in his body.

The key culmination of this analysis was that with the necessary genomic attributes, mentors, teachers, training opportunities, time to commit, the psychological commitment and drive, and money to afford all of this, portions of Batman’s mythology do resonate with reality.

In “Inventing Iron Man” I borrowed from Samuel Taylor Coleridge and asked readers to use a “willing sense of disbelief” and imagine that Iron Man’s exoskeletal suit of armor actually existed. Then I used the structure of the book to explore how such a suit of armor could actually work in connection with the body. The main focus is considering Iron Man as a biological control problem of human ability enhanced by technology (Zehr, 2011b).

Where Batman represents the ultimate in human conditioning by training, Iron Man becomes the ultimate in brain–machine interface. The suit of armor can also be considered a form of advanced “tool” for Tony Stark’s brain to use. Pathological outcomes are also raised when discussing the implications for adding another tool to the body schema. This brings out issues like phantom limbs and cortical plasticity in both beneficial and pathological outcomes, which were also explored in posts at *Scientific American* (Zehr, 2012a). Thus, much of the content in “Inventing Iron Man” centers on the engineering and neuroscience concepts contained in the rapidly expanding field of brain-machine interface.

In addition to extreme performance, superheroes can also be used as metaphors for “normal” and pathologically reduced performance. For example, in “Inventing Iron Man” I suggest that the habitual use of a real Iron Man exoskeleton would result in extensive physiological deconditioning effects and negative health implications. These examples are paralleled with real-world examples of deconditioning found after physical inactivity and long-term space flight.

The implications of concussion and mild traumatic brain injury were addressed in my first three books (Zehr, 2008, 2011b,2014b) and also in blogs (Zehr, 2012b). I was careful to highlight the dysfunction that occurs with increased energy demand and neuronal metabolism along with decreased supply and how that gives rise to concussion symptoms. This leads to discussions about secondary impact syndrome, the importance of protecting the brain from future concussive incidents, and how such exposure may lead to increased symptoms and susceptibility with lower impacts occurring over time.

I have not only focused on Batman and Iron Man, though. Using other superheroes I have explored the genetic regulation of human muscle strength via myostatin in considering Superman (Zehr, 2013b), tissue repair and enhancement after orthopedic injury when considering the “healing factor” of Wolverine (Zehr, 2013a), and considering the creation of neurological chimeras with enhanced hippocampal processing in the form of Rocket Racoon from “Guardians of the Galaxy” (Zehr, 2014c).

My biggest challenge, however, was writing a book for young adults. Project Superhero distills the main themes in “Becoming Batman” and “Inventing Iron Man” but puts them in a context for young adults (Zehr, 2014b). My first attempt to do this was an incomplete failure. I tried to take the content from my first two books and just write it using simpler language. My agent was quick to let me know that this would not be very interesting for young readers, and that I should do a bit more reading of my own in young adult fiction and nonfiction.

The first-person diary-style narrative was (and is) hugely popular with younger readers. Examples abound, but one of the most well known is the “Diary of a Wimpy Kid” series by Jeff Kinney. After reflecting on the needs of this new audience I was trying to reach out to, I decided to adopt the popular diary style but also chose to combine commentary from real people in creating a hybrid fiction/nonfiction book.

Project Superhero includes many of the concepts in neuroscience, physiology, martial arts, and nutrition I wrote about in “Becoming Batman” and “Inventing Iron Man,” but which were now recast in a format that was more accessible for a younger age group. This also required me to produce a fictional story and narrative arc in the book to follow my protagonist Jessie and her friends across their eighth grade year.

While this book was my most challenging, it has also been extremely rewarding. I have received many letters and e-mails from readers, but the review I treasure most is one posted on *Goodreads* by teen “Daniel” (Goodreads, 2016):

When I pick books up from the library, I usually sort them into three piles, based on how much I want to read them. This is one of the few books from the third (least want to read) pile that I gave 5 stars. I seriously loved this book. It is amazing, from interviews with actual heroes, to the facts it blends in, to the story that is amazing. The book is themed on superhero comics, but have not read any comics and I still grasped the story good. Jessie is a quiet introvert that loves reading superhero comics with her friends. When she starts 8th grade, she is ecstatic to hear about the Superhero Slam debate competition, but can she overcome a fear of public speaking to become her own superhero? Overall AMAZING book!

My next book, “Creating Captain America: The Possibility of Enhancing Our Evolution” will be published in 2017 (Zehr, 2017). This book completes the trilogy I began with “Becoming Batman” by considering how much we can now alter our own biology using science and engineering. This book has a much larger philosophical focus, and a major theme is considering what we will accept as a society when it comes to the functional abilities of “normal” humans, a concept I have also written about in several journals (Zehr, 2015b,c).

Others have used James Bond to explore chronic alcoholism (Johnson et al., 2013) and Star Wars (in the form of Sith Lord Darth Vader) to explore respiratory disease (Plovsing and Berg, 2014; Berg and Plovsing, 2016). Popular culture for science communication can be found in the excellent “Physics of Superheros” and “The Amazing Story of Quantum Mechanics” by James Kakalios (Kakalios, 2006, 2010), and “The Science of Superheros” by Lois Gresh and Robert Weinberg (Gresh and Weinberg, 2003).

Another great example of fusing popular culture with science—physics in this case—is “The Physics of Star Trek” by Lawrence Krauss (Krauss, 1995). In the foreword to “The Physics of Superheroes,” Krauss nicely captured the idea of using the popular culture middle ground with “…few things are more memorable than confronting one’s own misconceptions… if you want to reach out to understand popular misconceptions, then exploiting where we get our cultural perspectives from is a good place to start. And if that means borrowing from Superman, or Star Trek, I am all for it!”

Of course, it is not necessary that popular culture examples be drawn solely from comic books or science fiction, as in many of the above examples. Other examples might be using past or present sports figures, applying famous historical events (e.g., the moon landing or the Battle of Troy) or using current news stories (e.g., Zika virus, water treatment, and the Olympics; vaccines and vaccination). With a little thought and planning, almost anything can be used to make science fun and accessible.

## Effective communication is all about accessibility and fun

**Figure 2.**
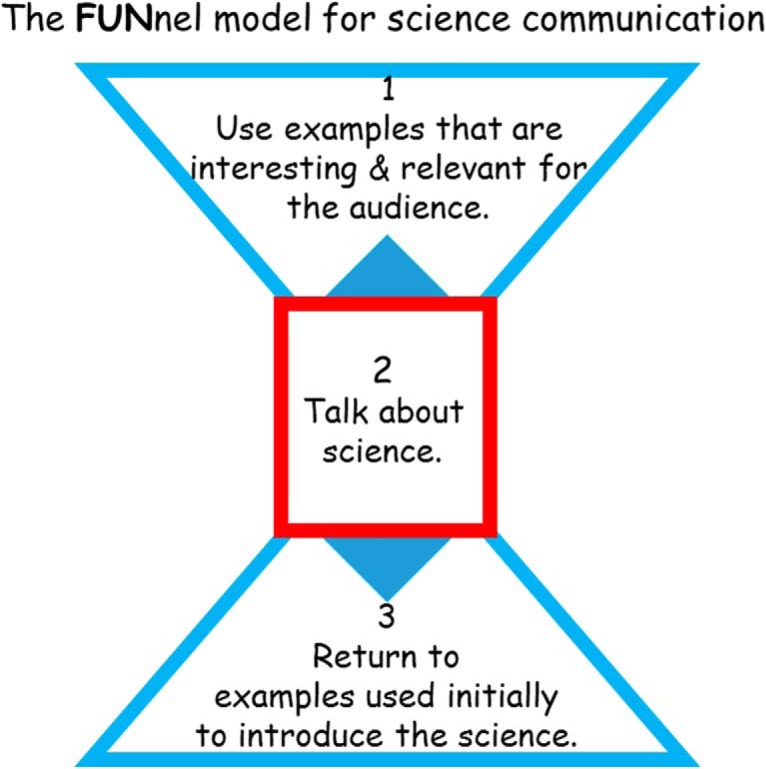
The FUNnel model for science communication. The simple concept here is to use popular culture as the lead-in and the summary for science concepts. For example, borrowing the approach I took in “Inventing Iron Man,” at Step 1 you might begin with Iron Man as a topical example of a human being with abilities amplified by technology. Then, asking how could this work, in Step 2 begin to lead into robotics, neuroprosthetics, and the brain–machine interface. This leads to a discussion about the organization of the brain and spinal cord, and how this allows for the ability to extract information about movement and movement planning that could be used to control an advanced prosthetic in the form of Iron Man. This leads into Step 3, where you return to the Iron Man armor and briefly summarize the level of technology currently available to support (or not) the comic book icon.

 In seeing the effectiveness of others’ science communication activities and reflecting on my own experiences in this area, I came to realize that the approach I took could be conceptualized as a dual funnel—wide at both ends and narrowing in the middle. The end portions represent engaging the general public with relevant examples, and the narrow bit in the middle is where all the science content finds a home. This is shown graphically as the FUNnel model for science communication in Figure 2.

## Our knowledge is power that we do not own

 I think we need to really ask, how effective do we actually want to be with science communication? Do we want to go through the motions of communicating or do we want to be truly effective? I suspect most of us do want to be effective and are intrinsically motivated to be so. If that is indeed true, then making all efforts to be maximally effective should be encouraged.

We scientists really do have a powerful role to play in our society. The science superpowers we possess include discovery, creation, synthesis, and dissemination of knowledge. For the last in that list, dissemination, it is critical to appreciate that this includes conversation with society at large, not just among our academic colleagues. That is the hidden science superpower that most of us have to work to realize. Yet, it is important work.

For we scientists to be effective, it is not sufficient to simply translate what we want to communicate into simpler words and concepts for nonspecialists. Instead, we need to put our concepts into the context the target audience is ready to receive. To be effective, this means going outside our comfort zones to more effectively enter the comfort zones of our audiences.

We need to go beyond simply asking what we think our audiences should need or ought to know. Instead, the real questions are how will they know it, what is the medium through which they are ready to know it, and how do I translate the scientific messages into a comfortable message for them?

There is a famous phrase from a very famous comic book written by Stan Lee and drawn by Steve Ditko. That comic book gave us the debut of a certain “Spider-Man” (*Amazing Fantasy* #15, published August 10, 1964), and in it Peter Parker learns an important lesson about responsibility. After failing to act to subdue a criminal, that criminal goes on to kill Parker’s Uncle Ben. Peter reflects that “…all my fault! If only I had stopped him when I could have! But I didn’t!” Stan Lee’s last words in the final panel of the comic states that “with great power there must also come—great responsibility!”

I propose that we all have our own superpowers of communication that we can use to stop misinformation and poorly conceived ideas that many may have about science because they are not engaging in our “traditional” outreach activities. We use our “powers” by stepping up and communicating with the other members of our communities and our society who are not scientists. Of course, to make increased advocacy activities sustainable in science, academic institutions and funding agencies must also demonstrate the value of these activities by tangible action.

To paraphrase Sir Frances Bacon, “knowledge is power.” It is time to accept that our efforts to create and generate scientific knowledge put great power into our hands. This also obliges us to exercise great responsibility. For that knowledge to have any value, it is our responsibility to affect the largest audience by communicating as widely as we possibly can.

The scientific knowledge we discover does not belong to us—we just had it first. We can honor that knowledge best by sharing it widely using the most creative means at our disposal.

## Synthesis

The decision was a result of the Reviewing Editor Anna Menini and the peer reviewers coming together and discussing their recommendations until a consensus was reached. A fact-based synthesis statement explaining their decision and outlining what is needed to prepare a revision is listed below. The following reviewers agreed to reveal their identity: Stuart Firestein

Paul Zehrs's article on science communication is a delightful read that does a first rate job of justifying the importance of science communication. Too often overlooked or looked down upon by established scientists Zehr provides not only reasons why this is an important activity but shows how a smart scientist can participate with the public while giving nothing up as a scientist - indeed I think while growing as a scientist. I applaud his efforts and this very readable and meaningful piece that I hope will influence younger scientists to take an interest in making their work accessible to the public. Erwin Schrodinger said, "If at the end of the day you cannot tell anyone about what you're doing, then your doing is worthless." Zehr lives up to that challenge.

My only comment might be that figure 1 is not really very explanatory and the cartoonish quality of it takes away from the seriousness of the argument being made. The article is already lighthearted enough in the writing style.
